# Cerebral thrombus analysis as a useful diagnostic tool for infective endocarditis in ischemic stroke patients

**DOI:** 10.1177/23969873251320449

**Published:** 2025-02-16

**Authors:** Aurora Semerano, Beatrice Dell’Acqua, Angela Genchi, Francesca Sanvito, Ghil Schwarz, Manuel Alejandro Montano Castillo, Andrea Bergamaschi, Michela Sampaolo, Erica Butti, Giorgia Serena Gullotta, Mariangela Piano, Marco Ripa, Paolo Scarpellini, Andrea Falini, Pietro Panni, Elio Clemente Agostoni, Nicola Clementi, Guillaume Saliou, Steven David Hajdu, Luisa Roveri, Patrik Michel, Gianvito Martino, Massimo Filippi, Davide Strambo, Marco Bacigaluppi

**Affiliations:** 1 Department of Neurology, IRCCS San Raffaele Hospital, Milan, Italy; 2 Neuroimmunology Unit, IRCCS San Raffaele Hospital, Milan, Italy; 3 Department of Pathology, IRCCS San Raffaele Hospital, Milan, Italy; 4 Neurology and Stroke Unit, ASST Grande Ospedale Metropolitano Niguarda, Milan, Italy; 5 Laboratory of Microbiology and Virology, IRCCS San Raffaele Hospital, Milan, Italy; 6 Neuroradiology Unit, ASST Grande Ospedale Metropolitano Niguarda, Milan, Italy; 7 Department of Infectious Diseases, IRCCS San Raffaele Hospital, Milan, Italy; 8 Department of Neuroradiology, IRCCS San Raffaele Hospital, Milan, Italy; 9 Department of Diagnostic and Interventional Radiology, University Hospital of Lausanne and University of Lausanne, Lausanne, Switzerland; 10 Stroke Center, Neurology Service, University Hospital of Lausanne and University of Lausanne, Lausanne, Switzerland

**Keywords:** Stroke etiology, infective endocarditis, thrombosis, pathogen detection

## Abstract

**Introduction:**

Infective endocarditis (IE) is a life-threatening condition and a rare cause of ischemic stroke (IS). This study aimed to evaluate the utility of analyzing cerebral thrombi, obtained through endovascular thrombectomy in IS, for the pathological diagnosis of IE.

**Patients and methods:**

Cerebral thrombi from three groups of IS patients were compared: definite IE (*n* = 10), cardioembolic stroke without and with concomitant infection (CE-I^−^: *n* = 30, CE-I^+^: *n* = 10). We performed histological examination, molecular biology, and microbiological tests on cerebral thrombi, to detect microorganisms and assess their composition.

**Results:**

Median age of included patients was 73 years and 50% were females. Hematoxylin & Eosin and Grocott-Gomori Methenamine Silver stains detected microorganisms in all IE cerebral thrombi, and none in the control groups. Thrombus PCR detected relevant microorganism in n = 2/7 IE. Compared to control groups, IE thrombi were characterized by significant lower content of red blood cells (median [IQR]: IE = 7.4 [4.2–26.7], CE-I^−^ = 49.3 [17–62.6], CE-I^+^ = 57.5 [40.7–60.8], % over thrombus section area [%TSA], *p* = 0.001), increased von Willebrand Factor (IE = 23.9 [19.1–32], CE-I^−^ = 11.2 [8.2–12.8], CE-I^+^ = 12.9 [10.7–18.3], %TSA, *p* = 0.001), cell-dominant pattern of Neutrophil Extracellular Traps (IE = 100%, CE-I^−^ = 69%, CE-I^+^ = 70%, *p* ⩽ 0.001), and more frequent sub-acute or chronic thrombus age classification (*p* ⩽ 0.001). These latter thrombus features displayed good discriminative ability between IE and controls, with AUC values between 0.84 and 0.95.

**Discussion:**

Multimodal analysis of cerebral thrombi in IS with suspected IE supports early and definite pathological diagnosis by detecting pathogens and assessing changes in thrombus composition.

## Graphical abstract

**Graphical Abstract:**
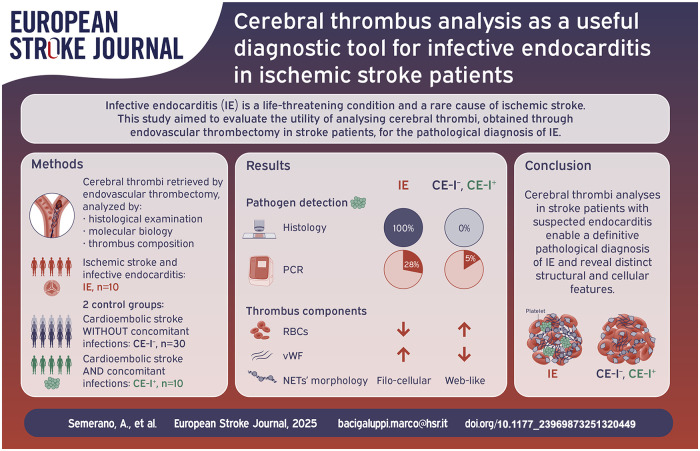
This is a visual representation of the abstract.

## Introduction

Over the past two decades, infective endocarditis (IE) has seen a significant increase in incidence and mortality,^1^ with neurological complications playing a major role.^2^ Ischemic strokes (IS) from vegetation embolization are common in IE, often presenting as initial symptoms.^3^ Prompt diagnosis and treatment are crucial to prevent embolization and reduce mortality,^4^ but it can be challenging especially in the acute stroke setting. Moreover, up to 30% of patients with IE present with negative findings on echocardiography or blood cultures.^5^

Endovascular thrombectomy (EVT), employed as acute treatment for large vessel occlusion strokes, allows the retrieval of embolized cerebral thrombi and their analysis for diagnostic purposes.^6^ The most recent 2023 Duke-International Society for Cardiovascular Infectious Diseases (ISCVID) IE diagnostic criteria underline the importance of the pathological analysis of arterial emboli for definite diagnosis ^7^; furthermore, 2023 European Society of Cardiology Guidelines for the management of IE first introduced that, when performing EVT, the retrieved embolic material should undergo pathological and microbiological analyses.^8^ However, the histological characteristics of thrombi that define IE in cases of cerebral embolization, as well as their diagnostic value in distinguishing them from non-IE cases, have not yet been clearly established.

In this study, we aimed (i) to assess the ability of histological and microbiological analyses to detect and identify microorganisms within cerebral thrombi and evaluate their diagnostic performance in the diagnosis of IE, and (ii) to characterize composition of cerebral thrombi in IE.

## Material and methods

### Study population

This study was conducted on consecutive acute IS patients admitted to three comprehensive stroke centers between April 2017 and September 2022 who were treated with EVT and had cerebral thrombi available for histological analysis. Patients’ clinical and radiological data were prospectively collected in the local hospital stroke registries.

For this study we selected:

(i) patients diagnosed with definite IE according to the modified Duke clinical criteria after a complete diagnostic work-up including blood cultures and transthoracic and/or transesophageal echocardiography (IE group);
(ii) a control group of patients diagnosed with cardioembolic stroke according to the TOAST criteria, with no evidence of any concomitant infection at the time of stroke onset (CE-I^−^ group), matched 1:3 with IE patients, for age, sex, intravenous thrombolysis, and previous anti-thrombotic treatment. In the matching procedure, we prioritized variables more likely to influence thrombus composition, such as IVT administration and pre-stroke anticoagulant use;
(iii) a second control group of consecutive patients in equal number to the IE group, with cardioembolic stroke according to TOAST criteria, and concomitant infection other than IE at the time of stroke onset (CE-I^+^ group). The presence of concomitant infection was determined based on clinical signs, symptoms, and additional diagnostic tests, including urinary and hematological counts, cultures, and imaging studies. The criteria for infection, adapted from previously established publications,^9^ required:
(1) At least one typical symptom or sign of infection, along with either a documented increase in body temperature (>37.5°C), a microbiological finding suggestive of acute infection, or a corresponding pathological finding on diagnostic examination (e.g. radiograph, urine culture, blood culture);
(2) a combination of two or more clinical symptoms commonly associated with localized infection.

Typical symptoms included sore throat with lymphadenitis, cough producing purulent sputum, vomiting, urinary urgency, hematuria, dysuria, diarrhea, pressure sores, otalgia, flu-like symptoms, papular or vesicular erythema, and ulcerated skin lesions. We classified the infection type as skin, urinary tract, septicemia, abdominal, or respiratory infection. Symptoms of infection had to be present within the week prior to ischemia. Positive infection status was excluded if the information was insufficient, or if there was an alternative, noninfectious explanation for the symptoms.

Due to the limited number of these patients, this control group was not matched with IE patients for clinical features, as meaningful matching would not have been feasible.

For each patient we recorded demographic data, vascular risk factors, therapy at stroke onset, clinical history, imaging and procedural data, stroke etiology, laboratory values within 24 h from stroke onset, and 3-month modified Rankin Scale. Presentation of IE was considered subacute if signs and symptoms had started 1–6 months prior to diagnosis, and acute when signs and symptoms had started less than 1 month prior to diagnosis.

### Cerebral thrombi analysis

#### Material collection and processing

Immediately after retrieval during EVT, cerebral thrombi were fixed in 10% formalin and stored at +4°C until processing. Formalin-fixed specimens were then longitudinally embedded in paraffin and cut in serial sections of 5 μm. Thrombus analysis was centrally performed at one of the participating centers. For each analysis multiple sections for each thrombus were analyzed (2–4 sections per thrombus per staining) to account for the heterogeneity of the thrombus.^10^

#### Microorganism detection

Thrombi were stained with hematoxylin and eosin (H&E), Grocott’s methenamine silver (GMS), GRAM and/or Periodic Acid Schiff (PAS) staining and evaluated at 63× or higher magnification by an expert pathologist, to visually detect the presence of bacteria or fungi.^11,12^ When analyzing samples for this study, the pathologist was informed about the clinical suspicion of endocarditis but was blinded to the final diagnosis.

For molecular diagnostics, polymerase chain reaction (PCR) amplification of 16S-rDNA followed by Sanger sequencing was performed. For the PCR analysis, we extracted total DNA from paraffin-embedded thrombotic material using the QIAamp DNA FFPE Tissue Kit (Qiagen), following the manufacturer’s instructions. To obtain a viable DNA yield, we processed at least 10–15 sections, each 7 µm thick. However, in cases where thrombi were small or the available material was insufficient, priority was given to histological analysis, and amplification was not performed. DNA concentration and quality were measured. Complete details on the metagenomic analysis process are described in the Supplemental Methods.

In two patients with IE highly suspected since hospital admission, we were able to perform a bacterial culture on a fresh fragment of the cerebral thrombus. Freshly extracted thrombi were aseptically dissected and a portion set aside for culturing.

#### Thrombus composition

We adopted Martius Scarlet Blue (MSB) staining to assess the content of red blood cells (RBCs), fibrin, and platelets/other thrombus components, identified by yellow, pink, and gray coloration, respectively.^13^ Immunohistochemical staining was further performed to assess platelet (CD61^+^ areas; anti-CD61, 1:100, Dako), von Willebrand Factor (vWF) (vWF+ areas; anti-vWF, 1:1000, Abcam, ab6994), and neutrophil extracellular traps (NETs) composition (citH3^+^ areas; anti-citH3, 1:200, Abcam). Stained sections were scanned with Aperio^®^ Microscope Digitizer (Leica Biosystems), at 20× magnification. Areas of RBCs, fibrin, platelets, vWF, and NETs were automatically quantified using the classification algorithm of Orbit Image Analysis^®^ (v3.64) Software. The quantification was performed by measuring the percentage of positively stained area relative to the total area on each section, then calculating an average across the various sections analyzed within each thrombus. The morphological features of NETs were visually assessed, and thrombi were categorized into cell-dominant or web-dominant pattern, as previously described.^14^ In the cell-dominant pattern, NETs remain closely associated with the neutrophil cell structure, while in the web-dominant pattern, NETs form extensive web-like structures, spreading out into the extracellular space. These patterns are thought to reflect differences in the underlying biological processes of NET secretion, potentially influenced by the type or intensity of the inflammatory trigger.^15^

We also assessed the histological age of the thrombus based on previously established criteria,^16^ initially developed for deep venous thrombosis, and adapted for the analysis of cerebral thrombi. As a result, we classified thrombi into three progressive time phases according to the prevalent pattern. Phase 1 (acute) thrombi are characterized by platelet plugging, fibrin deposition with a layered growth (Zahn’s lines), and preserved and agglomerated RBCs. In phase 2 (subacute), macrophages containing hemosiderin predominate, RBCs ghosts, nuclear debris of leukocytes, and fibrinous transformation are evident. In phase 3 (chronic), the thrombi became hyalinized, and few leukocytes are visible between compact, fiber-rich connective tissue.

### Statistical analysis

We summarized continuous variables using median values with interquartile range (IQR) and presented categorical variables as absolute numbers and percentages. We compared clinical and thrombus component variables between IE, CE-I^−^ and CE-I^+^ groups using Fisher’s exact test for categorical variables and Kruskall-Wallis test for continuous variables, as appropriate. Pairwise comparisons between the IE group and the two control groups were corrected for multiple comparisons using Dunnett’s method.

*p*-Values < 0.05 were considered statistically significant. For thrombus components significantly associated with IE in univariable analysis, we built a receiver operating characteristic curve (ROC) using the thrombus component as predicting variable and IE as response variable. Discrimination of each variable was assessed by calculation of the area under the ROC (AUC).

Statistical analyses were performed with R statistical software (version3.3.2, R Core Team [2016]).

## Results

### Clinical characteristics

From 437 consecutive patients who underwent EVT with a cerebral thrombus retrieved and available for analysis across the three centers during the study period, we identified 10 patients with a diagnosis of definite IE according to the modified Duke clinical criteria.^7^ During the study period, other 10 IS patients met the Duke criteria for IE but were excluded as they did not undergo EVT (*n* = 8) or thrombus was not retrieved and not available for analysis (*n* = 2). (A flowchart displaying the selection process in Supplemental Figure 1). A group of 30 matched patients with cardioembolic stroke and no concomitant infection (CE-I^−^ control group) and 10 patients with CE stroke and non-endocarditic concomitant infection (CE-I^+^) were included as control groups. The median age of the study population was 73.7 years, 50% were females, and median baseline NIHSS was 15 points. Clinical and radiological characteristics of the three groups and details are reported in Supplemental Table 1. Details regarding the clinical presentation and the diagnosis of IE are reported in Supplemental Table 2. In the CE-I^+^ group, the site of infection was pulmonary in four patients, urinary in four, gastrointestinal in one, and undetermined in one patient; on admission, five patients had a fever, nine elevated CRP and four exhibited leukocytosis; blood cultures were performed in four patients, all of which were negative.

### Detecting the pathogen on thrombus specimen

**Figure 1.**
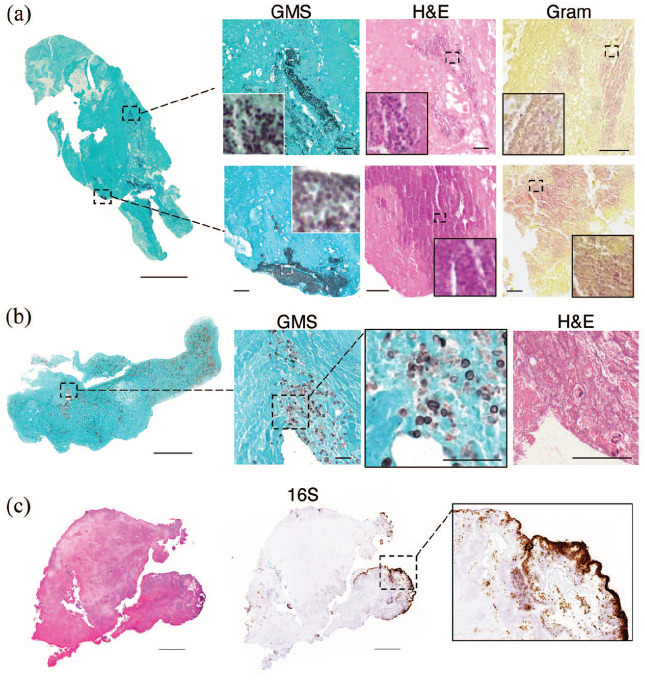
Microorganism detection on cerebral thrombi from IE patients: (a) low magnification of a cerebral thrombus from a patient with bacterial IE, stained by GMS. Higher magnification images on the right show sites of bacterial localization after GMS staining (black spots), H&E (dark pink spots) and Gram (pink spots). (b) Low magnification of a cerebral thrombus from a patient (different from the patient shown in (a)) with fungal IE, stained by GMS. In higher magnification, yeast forms can be identified on GMS (dark spots) and H&E. (c) Low magnification of a cerebral thrombus from another patient with bacterial IE after H&E, and in situ hybridization for the bacterial rRNA 16s. In the higher magnification, positive 16s areas, representing bacterial colonies (brown), are mostly distributed near thrombus surface. Scale bars: 600 and 30 µm.

Histopathological stain with H&E and/or GMS detected microorganisms in all thrombi within the IE group, and none in the two control groups. In most of IE thrombi (9 of 10), we observed cocci arranged heterogeneously within the thrombus in clusters and chains that could also be visualized on GMS or on GRAM staining (Figure 1(a) and (c)). In the remaining IE case (1 of 10), GMS and PAS staining allowed instead the detection of clusters of yeast forms and pseudo hyphae with a diagnosis of fungal endocarditis (Figure 1(b)). In situ hybridization for 16S bacterial rRNA provided additional confirmation of the microbial presence within thrombi (Figure 1(c)).

Pan-bacterial 16S PCR analysis on the thrombotic material was performed for 7 out of 10 patients in the IE group and 29 out of 40 patients in the two control groups (7 in CE-I^+^ and 22 in CE-I^−^). For the remaining patients, PCR amplification was not performed due to an insufficient quantity of material. Thrombus PCR was positive in four out of seven patients in the IE group and in one out of 29 patients in the control groups. In the latter case, from the CE-I^−^ group, the pathogen detected by sequencing the PCR product was *Cutibacterium acnes*, which was considered as a contamination. The details of microorganisms detected through sequencing of PCR amplifications on cerebral thrombi, thrombus and blood cultures in each IE patient are shown in Table 1. Among the four IE patients with positive PCR in cerebral thrombi, one also had positive hemocultures for the same pathogen (*Streptococcus gordonii*, case #5), and another had negative hemocultures but a concordant result on thrombus culture, which was positive for *Streptococcus gallolyticus* (case #6). In the other two patients, the thrombus metagenomic sequencing analysis revealed a pathogen not concordant with blood cultures: in case #7, the thrombus metagenomics revealed *Streptococcus spp*, while blood cultures were positive for *Staphylococcus aureus*. This patient was known for a previous, clinically resolved streptococcal endocarditis 8 months earlier, suggesting the possibility of residual DNA material from the previous infection that could explain the PCR result.^17^ In case #4, the thrombus metagenomics sequencing revealed *Pseudomonas spp* while blood cultures were positive for *Streptococcus mitis*. We interpreted the finding in the thrombus as potentially due to preanalytical contamination, given the environmental nature of certain Pseudomonadaceae species, which cannot be excluded. Overall thrombus 16S PCR followed by Sanger sequencing from paraffin-embedded thrombotic material had low sensitivity (28%), but high specificity (96%) for identifying pathogens responsible for infective endocarditis (Table 2).

**Table 1.** Comparative summary of results from thrombus histopathology, PCR, and blood cultures.

Thrombus	Cardiac valve	Blood cultures	Thrombus histology	Thrombus PCR	Thrombus culture
*#01*	Aortic bioprosthetic valve	*Candida parapsilosis*	Positive (Hyphae)	np	np
*#02*	Native mitral valve with prosthetic ring annuloplasty	*Staphylococcus aureus*	Positive (Cocci)	np	np
*#03*	Mechanical prosthetic aortic valve	*Streptococcus dysgalactiae*	Positive (Cocci)	np	np
*#04*	Native aortic valve	*Streptococcus mitis*	Positive (Cocci)	*Pseudomonas spp*	np
*#05*	Mechanical prosthetic aortic valve	*Streptococcus gordonni*	Positive (Cocci)	*Streptococcus spp*	np
*#06*	Native mitral valve with prosthetic ring annuloplasty	Negative	Positive (Cocci)	*Streptococcus spp*	*Streptococcus gallolyticus*
*#07*	Mechanical prosthetic mitral valve	*Staphylococcus aureus*	Positive (Cocci)	*Streptococcus spp*	np
*#08*	Mechanical prosthetic aortic valve	*Streptococcus epidermidis*	Positive (Cocci)	Negative	np
*#09*	Native mitral and aortic valves	*Enterococcus faecalis*	Positive (Cocci)	Negative	np
*#10*	Native mitral valve	*Streptococcus gallolyticus*	Positive (cocci)	Negative	*Staphylococcus epidermidis*

MSSA: methicillin-sensitive *Staphylococcus aureus*; np: not performed/not available.

**Table 2.** Diagnostic performances of thrombus histology and thrombus PCR in detecting pathogens responsible of infective endocarditis.

Variable	TP	FP	TN	FN	Sensitivity	Specificity	PPV	NPV	Accuracy
Pathogen detection on thrombus histology	10	0	40	0	100% (69.2%–100%)	100% (91.2%–100%)	100% (69.2%–100%)	100% (91.2%–100%)	100% (92.9%–100%)
Thrombus PCR	2^a^	1	28	5^b^	28.6% (3.7%–71%)	96.6% (82.2%–99.9%)	66.7% (9.4%–99.2%)	84.8% (68.1%–94.9%)	83.3% (67.2%–93.6%)

TP: true positives; FP: false positives; TN: true negatives; FN: false negatives; PPV: positive predictive value; NPV: negative predictive value.

a Cases in which PCR successfully amplified a pathogen corresponding to the etiological agent of IE.

b No amplification, or amplification of a pathogen not corresponding to the designated etiological agent in patients with IE.

### Structure, immune phenotype, and age of cerebral thrombi in stroke patients with endocarditis

For the analysis of clot composition, we excluded the sample from a patient in the IE group who had concomitant COVID-19, due to the potential alteration of the structural and immune cell composition of the thrombus as previously described.^18^

**Figure 2.**
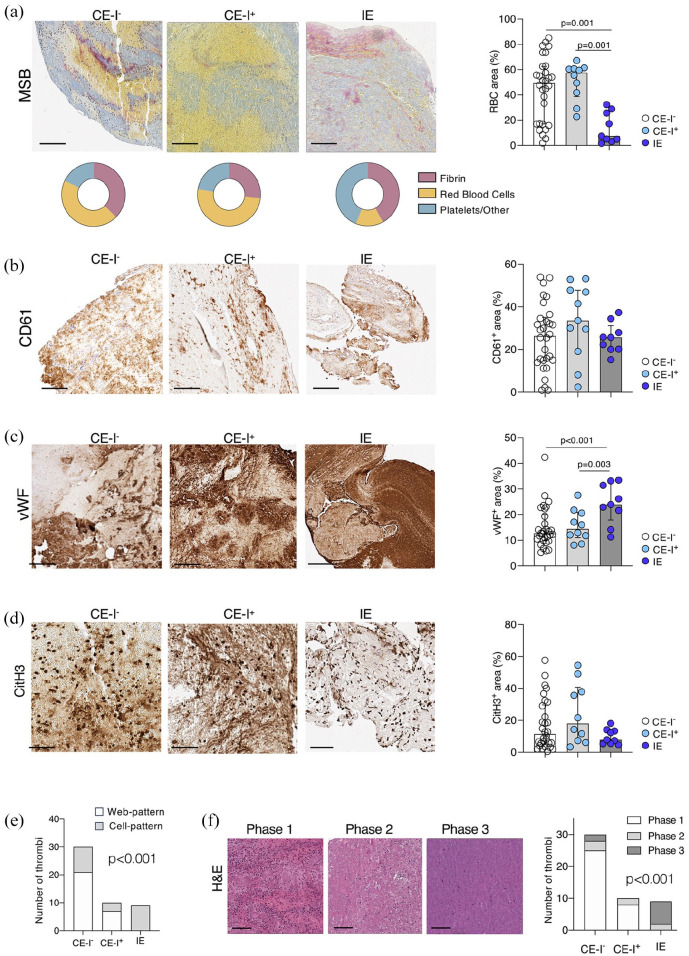
Composition of cerebral thrombi of patients with IE and controls: from left to right, representative images of cerebral thrombi and scatterplot displaying the quantification of RBCs (MSB-yellow^+^) (a), platelets (CD 61^+^) (b), vWF^+^ (c), and NETs (citH3^+^) (d) % over thrombus section area in the three patients’ groups (*n* = 30 CE-I^−^, *n* = 10 CE-I^+^ and *n* = 9 IE). In the scatterplots, superposed bars height indicates median value in the group and whiskers the IQR. *P*-values of pairwise comparisons (Dunnett’s adjustment) are reported when significant. In (a) the donut plot displays the distribution of the three MSB components. (e) Distribution of the NETs pattern (cell pattern and web-pattern) in the three patients’ groups, chi-square test, *p* ⩽ 0.001. (f) From left to right, representative H&E images of cerebral thrombi in different aging-phases (from Phase 1 to Phase 3) and distribution of the age-phases of the thrombus in each study group (CE-I^−^, CE-I^+^, IE), chi-square test, *p* ⩽ 0.001. Scale bars in (a–c and f) 200 μm; in (d) 100 µm.

Endocarditic thrombi had markedly reduced RBCs content compared to control groups (49.3 [17–62.6], 57.5 [40.7–60.8], 7.4 [4.2–26.7], median [IQR], in CE-I^−^, CE-I^+^, and IE respectively, MSB-yellow^+^ percentage over thrombus section area [%TSA], *p* = 0.001, Figure 2(a)). Fibrin and platelets content was similar in the three groups (Figure 2(a) and (b)), while thrombi of IE patients significantly had higher vWF density (11.2 [8.2–12.8], 12.9 [10.7–18.3] and 23.9 [19.1–32] in CE-I^−^, CE-I^+^, and IE, respectively, vWF^+^ %TSA, *p* = 0.001; Figure 2(c)). The density of NETs did not significantly change in thrombi from the different groups (Figure 2(d)), however, the pattern of NETs was different. Endocarditic thrombi had a prevalent cell-dominant NET pattern while the control groups were rather characterized by a web-dominant NET pattern (30%, 30%, and 100% in CE-I^−^, CE-I^+^, and IE, respectively, % of cell-pattern, *p* < 0.001, Figure 2(e)).

The analysis of thrombus histological phase revealed that in the two control groups thrombi were mainly of recent formation, with 82.5% of thrombi classified as acute thrombotic process. Compared to controls, thrombi from stroke patients with endocarditis had a significantly different distribution of thrombus age classification (*p* < 0.001), as they resulted with sub-acute (22.2%) or chronic (77.8%) characteristics (Figure 2(f)).

**Figure 3.**
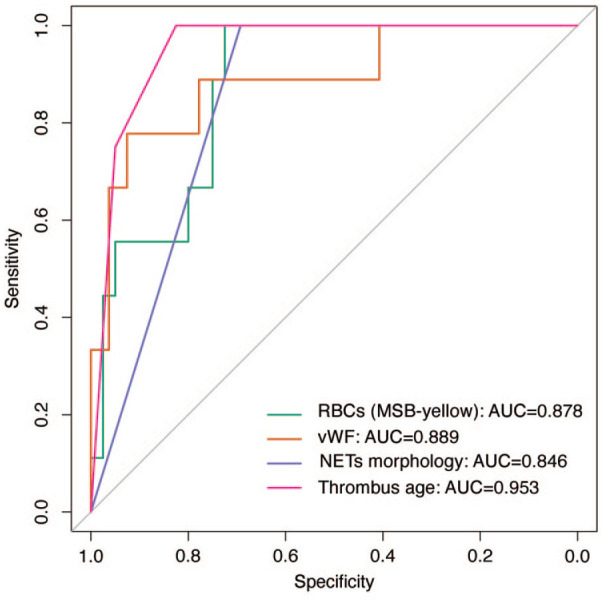
ROC curve analysis: the ROC curve analysis evaluating the performance of three thrombus components (RBC, vWF, NETs morphology), and thrombus age in discriminating infective endocarditis (IE) and control thrombi.

In the ROC curve analysis, the three thrombus components that exhibited a significant difference between IE and control thrombi in the univariable comparison (RBC, vWF, NETs morphology), along with thrombus histological age, displayed good AUC values, ranging between 0.846 and 0.953, in discriminating infective endocarditis (IE) and control thrombi. (Figure 3).

## Discussion

In this study on patients with stroke and IE, we found that: (i) histopathological stains successfully detected microorganisms within cerebral thrombi from IE; (ii) bacterial PCR of cerebral thrombi may provide valuable supplementary diagnostic information in selected cases; (iii) cerebral thrombi from IE patients exhibited distinctive features, including lower red blood cell, increased vWF content, a higher prevalence of NETs with a cell-dominant pattern, and hallmarks of more advanced age.

The identification of bacteria or fungi within thrombotic material using various techniques is of paramount importance, as it allows us to meet the pathological criteria for definite endocarditis according to the 2023 Duke-ISCVID IE Criteria.^7^ It is important to consider that up to 30% of patients with infective endocarditis (IE) present with negative findings on echocardiography or blood cultures. Therefore, having an additional pathological tool, such as the analysis of an arterial thrombus, could be invaluable in reaching the definite diagnosis of endocarditis.

While previous case reports and small case series have described the detection of microorganisms in cerebral thrombus analysis in patients with infective endocarditis, with findings similar to ours^19–26^ this is the first study to systematically evaluate the contribution of multi-parametric thrombus analysis to the pathological diagnosis of infective endocarditis. The methods we employed to detect pathogens on cerebral thrombi, including histological analysis and PCR, have been previously adopted in studies on cardiac valves.^27^ On cardiac valves, histopathology demonstrated 100% specificity for IE and showed good sensitivity. In our study, histological analysis of cerebral thrombi showed comparable diagnostic performance, with 100% sensitivity and specificity, although these findings should be interpreted cautiously due to the limited sample size. This technique allows for direct visualization of pathogens, but it has the limitation of not providing classification or identification of the exact responsible microorganism. Nevertheless, the direct visualization of bacteria or fungi in thrombus specimens leads to a definite diagnosis of endocarditis and support the initiation of empiric antibiotic therapy.^7^ It is important to emphasize that the effective application of this technique relies on the expertise of a pathologist who carefully examines the specimens and employs various histological staining methods to confirm the presence of pathogens. However, the staining techniques for direct visualization of bacteria or fungi, along with pathologist training, could be effectively implemented as part of routine diagnostics in most pathology departments. When available, supplementary organism-specific immunohistochemical stains can be an additional useful tool for refining pathogen identification.^28^

The second detection method, 16S rDNA PCR followed by sequencing, demonstrated lower sensitivity in detecting the bacterial pathogen responsible for IE compared to histopathological analysis. Nevertheless, 16S-PCR and sequencing demonstrates very high specificity (>90% in our study) and potentially offers an important advantage: in addition to detecting the presence of bacteria in the thrombus, it can, when successful, also identify the specific bacteria involved. This may be particularly valuable for identifying pathogens that are difficult to culture using current methods.^29^ Despite this potential advantage, PCR-based identification has limitations, as previously noted in studies on surgical heart samples.^30^ Our results further highlight its reduced sensitivity and specificity, possibly due in part to the extraction of genomic material from paraffin-embedded thrombi, which limits the tissue available for analysis. Of note, all the sequencing analyses have been carried out through Sanger based sequencing, which does not always allow microbial identification in case of mixed microbial DNA population. Additionally, and more importantly, sequencing results should be always interpreted cautiously and carefully related to clinical findings due to the risk of sampling contamination during the pre-analytic phase, which can lead to false positive identifications. Furthermore, in patients with previously treated IE, bacterial DNA may persist on thrombotic material, potentially leading to discordant results. Despite these limitations, 16S sequencing on thrombotic material can still provide a definite pathological diagnosis of endocarditis.^7^

Additional analysis, such as thrombus culture, can help identify the sensitivity profile of the IE-related pathogen. However, it is worth noting that studies on valve culture in infective endocarditis have reported relatively low sensitivity, ranging from 13% to 25%.^31^ The administration of antibiotic therapy prior to the cultural examination plays a significant role in explaining these findings, as the sensitivity of growth on valve culture has been inversely correlated with the duration of antibiotic treatment.^32^

In addition to the presence of bacteria, in our study thrombi from IE patients exhibited distinct characteristics in term of clot composition when compared to control samples. The different clot composition observed in IE patients may be attributed to the mechanisms of thrombus formation and inflammation, partly studied in models of Staphylococcus-induced endocarditis.^33^ The increased content of vWF in IE thrombi likely reflects the mechanisms of thrombus formation in endocarditis that may initiate from an underlying endothelial inflammation; indeed, vWF was abundant in induced endocarditis lesions and proved to be crucial in *S. aureus* adhesion to both damaged and inflamed hearth valves in mice.^33^ Moreover bacteria can per se trigger the release and bind vWF^34^ thus possibly explaining our observation. The lower content of RBCs in IE compared to cardioembolic thrombi could in part be attributed to the age disparity of the thrombi, as endocarditic thrombi were also found to be older, and it is possible that RBC degradation occurs over time.^35^ The finding of predominant cellular-like pattern of NETs deposition in IE thrombi might rely on a diverse modality of secretion,^15^ or on an early bacteria-related digestion of secreted NETs to escape from the host’s defense mechanisms.^36^

Based on our case series, we propose a protocol, outlined in the flowchart shown in Supplemental Figure 2, to guide the management of thrombus material in the angiographic suite following removal of the occluding thrombus. When clinical suspicion of endocarditis is present at the time of thrombectomy, the retrieved thrombus should be promptly sent to the pathology department for histopathological analysis and microbiology department in a sterile tube for microbial culture (possible only on freshly collected tissues). Conversely, in cases where there is no initial suspicion of endocarditis, we suggest routinely preserving the clot in paraffin, allowing for future analysis if endocarditis becomes a concern during the clinical workup after thrombectomy. While it is beyond the scope of our study, routinely retaining clots for analysis could offer valuable diagnostic insights beyond endocarditis, potentially aiding in the identification of rare stroke etiologies. Although beneficial in only a minority of cases, previous studies have shown that clot analysis can help identify various rare causes of ischemic stroke, further underscoring its clinical value.^37–39^

In this study, the thrombus analysis results were not routinely available to the clinical teams, as we conducted this research in an exploratory, observational setting without prioritizing prompt reporting. However, in a clinical context, the histological analysis can typically be completed within 24–72 h, with a similar timeframe required for fungal/bacterial metagenomics. Importantly, analysis of cerebral thrombi in cases of suspected endocarditis should not be considered a substitute for standard diagnostic workup including blood cultures and echocardiography. Instead, it should be regarded as a complementary, valuable tool to achieve a definite pathological diagnosis of IE. Given that histology and metagenomic analyses can be completed in a relatively short time, they may provide valuable information within a timeframe useful to support effective clinical decision-making.

The main limitations of this study include the relatively small number of endocarditis thrombi analyzed, which may limit the generalizability of our findings. Additionally, we could not perform a direct comparison between cerebral thrombi and valve histology, which could have provided further insights. Another limitation is the absence of control patients with positive blood culture in the CE-I^+^ group, which would the ideal comparator for patients with IE. While no such patients were present in our cohort, our adoption of rigorous criteria for defining concomitant infections, based on objective findings beyond symptoms alone, suggested a high likelihood of bacterial origin in patients in the CE-I^+^ group, and represents the best available representation of concomitant infection within our sample. We also acknowledge that not all patients with IS and suspected IE undergo EVT, nor is a cerebral thrombus always available for analysis. However, this still represent a relevant number of patients, considering that approximately 1–1.5 million cases of endocarditis are diagnosed annually, around 200,000 of these patients experience a stroke, and about 40% of them present with a large artery occlusion.^40^ Further, we recognize that the accessibility of thrombectomy procedures, and consequently the feasibility of using thrombus analysis for establishing the pathological diagnosis of IE, can be impacted by logistical challenges and cost considerations. Access to thrombectomy varies significantly worldwide, which may impact the consistency and practicality of using thrombus analysis as a diagnostic aid for IE on a broader scale. Even though our results apply only to this subset of patients, when a cerebral thrombus is available, its multi-parametric pathological assessment—including histological analysis and PCR/sequencing,—provides valuable information for the definite pathological diagnosis and for guiding antibiotic therapy. Further research into cost-effective and scalable methods for thrombus preservation and analysis could make these diagnostic insights more accessible in the future. While larger studies are needed to validate our findings, we suggest that thrombus analysis should be considered whenever embolized material is available in patients with suspected IE, as histology offers high sensitivity and specificity for IE diagnosis, and molecular amplification techniques or thrombus culture can provide microorganism-specific information.

## Supplemental Material

sj-docx-3-eso-10.1177_23969873251320449 – Supplemental material for Cerebral thrombus analysis as a useful diagnostic tool for infective endocarditis in ischemic stroke patients Supplemental material, sj-docx-3-eso-10.1177_23969873251320449 for Cerebral thrombus analysis as a useful diagnostic tool for infective endocarditis in ischemic stroke patients by Aurora Semerano, Beatrice Dell’Acqua, Angela Genchi, Francesca Sanvito, Ghil Schwarz, Manuel Alejandro Montano Castillo, Andrea Bergamaschi, Michela Sampaolo, Erica Butti, Giorgia Serena Gullotta, Mariangela Piano, Marco Ripa, Paolo Scarpellini, Andrea Falini, Pietro Panni, Elio Clemente Agostoni, Nicola Clementi, Guillaume Saliou, Steven David Hajdu, Luisa Roveri, Patrik Michel, Gianvito Martino, Massimo Filippi, Davide Strambo and Marco Bacigaluppi in European Stroke Journal

sj-pdf-1-eso-10.1177_23969873251320449 – Supplemental material for Cerebral thrombus analysis as a useful diagnostic tool for infective endocarditis in ischemic stroke patients Supplemental material, sj-pdf-1-eso-10.1177_23969873251320449 for Cerebral thrombus analysis as a useful diagnostic tool for infective endocarditis in ischemic stroke patients by Aurora Semerano, Beatrice Dell’Acqua, Angela Genchi, Francesca Sanvito, Ghil Schwarz, Manuel Alejandro Montano Castillo, Andrea Bergamaschi, Michela Sampaolo, Erica Butti, Giorgia Serena Gullotta, Mariangela Piano, Marco Ripa, Paolo Scarpellini, Andrea Falini, Pietro Panni, Elio Clemente Agostoni, Nicola Clementi, Guillaume Saliou, Steven David Hajdu, Luisa Roveri, Patrik Michel, Gianvito Martino, Massimo Filippi, Davide Strambo and Marco Bacigaluppi in European Stroke Journal

sj-pdf-2-eso-10.1177_23969873251320449 – Supplemental material for Cerebral thrombus analysis as a useful diagnostic tool for infective endocarditis in ischemic stroke patients Supplemental material, sj-pdf-2-eso-10.1177_23969873251320449 for Cerebral thrombus analysis as a useful diagnostic tool for infective endocarditis in ischemic stroke patients by Aurora Semerano, Beatrice Dell’Acqua, Angela Genchi, Francesca Sanvito, Ghil Schwarz, Manuel Alejandro Montano Castillo, Andrea Bergamaschi, Michela Sampaolo, Erica Butti, Giorgia Serena Gullotta, Mariangela Piano, Marco Ripa, Paolo Scarpellini, Andrea Falini, Pietro Panni, Elio Clemente Agostoni, Nicola Clementi, Guillaume Saliou, Steven David Hajdu, Luisa Roveri, Patrik Michel, Gianvito Martino, Massimo Filippi, Davide Strambo and Marco Bacigaluppi in European Stroke Journal
