# Supplementary material for: Cerebral thrombus analysis as a useful diagnostic tool for infective endocarditis in ischemic stroke patients
Source: Eur Stroke J. 2025 Feb 16;10(3):929–39. doi: 10.1177/23969873251320449 (PMC11831614; doi:10.1177/23969873251320449)
Supplement: sj-docx-3-eso-10.1177_23969873251320449 – Supplemental material for Cerebral thrombus analysis as a useful diagnostic tool for infective endocarditis in ischemic stroke patients [file sj-docx-3-eso-10.1177_23969873251320449.docx]

**Cerebral Thrombus Analysis as a Useful Diagnostic Tool for Infective Endocarditis in Ischemic Stroke Patients**

**SUPPLEMENTAL MATERIAL**

**Supplemental methods**

Polymerase chain reaction (PCR) amplification of the bacterial-specific 16S rDNA was performed using universal bacterial 16S rRNA primer set (16SFW 5’GATTAGATACCCTGGTAGTCCAC and 16SRW 5’ TACCTTGTTACGACTT ,). *E. coli* of a pre-existing positive sample was used as a positive control and nuclease-free UV-treated water as a negative control. The PCR was performed in a ProFlex PCR System (ThermoFisher Scientific). To detect PCR inhibitions and failures in DNA extraction and to avoid false-negative results, primers bGloF and bGloR (to detect human b-globin gene) were used for each sample in the same PCR conditions. PCR results were considered valid if all controls were negative or positive as appropriate and the b-globin gene was detected in all samples. We analyzed PCR products by electrophoresis on 1.5% agarose gels. PCR products displaying a band in the expected ~600-700 base pair region were directly sequenced with Sanger method using the same PCR primers. The samples were prepared using BigDye Terminator v.3.1 cycle sequencing kit (Applied Biosystem, Foster City, CA, USA) followed by a purification step with BigDye Xterminator™ Purification kit (Applied Biosystem, Foster City, CA, USA). We analyzed purified products on an automatic sequencer ABI PRISM 3730 genetic analyzer DNA Sequencer (Applied Biosystem, Foster City, CA, USA). The generated electropherograms were analyzed with SeqScape® Software (ThermoFisher Scientific, Waltham, MA, USA) and were then compared using the BLAST program available at the National Center for Biotechnology (<http://www.ncbi.nlm.nih.gov>). DNA sequences were further analysed by using EzBioCLoud ([www.ezbiocloud.net](http://www.ezbiocloud.net)) to provide a definitive identification of the organism to the species- or genus-level.

**Supplemental Table 1.** Characteristics of the study populations**.**

|  | **Total (n=50)** | **Infective endocarditis (n=10)** | **Cardioembolic w/o infection (n=30)** | **Cardioembolic with infection (n=10)** | **CE-I-  vs.   IE** | **CE-I+  vs.   IE** |
| --- | --- | --- | --- | --- | --- | --- |
| *Demographic data* |  |  |  |  |  |  |
| Age | 73.7 (65.9-80) | 66 (62.3-75.8)^+^ | 76.7 (70.8-81.1) ^+^ | 73 (66.7-80.6) | 0.100 | 0.212 |
| Sex | 25 (50%) | 7 (70%)^+^ | 16 (53.3%)^+^ | 2 (20%) | 0.530 | 0.047 |
| *Cardiovascular risk factors* |  |  |  |  |  |  |
| Hypertension | 38 (76%) | 6 (60%) | 24 (80%) | 8 (80%) | 0.336 | 0.468 |
| Diabetes | 9 (18%) | 2 (20%) | 5 (16.7%) | 2 (20%) | 0.958 | 1.000 |
| Dyslipidemia | 26 (52%) | 5 (50%) | 16 (53.3%) | 5 (50%) | 0.975 | 1.000 |
| Coronary artery disease | 12 (24%) | 1 (10%) | 8 (26.7%) | 3 (30%) | 0.457 | 0.471 |
| Smoking | 6 (12%) | 2 (20%) | 2 (6.7%) | 2 (20%) | 0.421 | 1.000 |
| Previous stroke or TIA | 10 (20%) | 3 (30%) | 5 (16.7%) | 2 (20%) | 0.559 | 0.794 |
| Atrial fibrillation | 32 (64%) | 3 (30%) | 23 (76.7%) | 6 (60%) | 0.014 | 0.244 |
| *Antithrombotic therapy at stroke onset* |  |  |  |  |  |  |
| None | 17 (34%) | 1 (10%)^+^ | 11 (36.7%)^+^ | 5 (50%) | 0.209 | 0.108 |
| Antiplateles | 11 (22%) | 4 (40%)^+^ | 4 (13.3%)^+^ | 3 (30%) | 0.139 | 0.796 |
| Anticoagulants | 22 (44%) | 5 (50%)^+^ | 15 (50%)^+^ | 2 (20%) | 1.000 | 0.294 |
| *Acute stroke treatment* |  |  |  |  |  |  |
| Intravenous thrombolysis | 3 (6%) | 0 (0%)^+^ | 0 (0%)^+^ | 3 (30%) | 1.000 | 0.005 |
| Onset-to-groin delay (minutes) | 255 (157-376.7) | 241 (167.9-295.8) | 255 (135-440) | 241 (195.1-298.8) | 0.614 | 0.999 |
| Baseline ASPECTS | 8.5 (8-10) | 7.5 (6-10) | 8 (8-9.6) | 10 (7.8-10) | 0.245 | 0.098 |
| *Occlusion site* |  |  |  |  |  |  |
| ICA | 10 (20.8%) | 2 (20%) | 6 (21.4%) | 2 (20%) | 0.993 | 1.000 |
| MCA | 33 (68.8%) | 8 (80%) | 18 (64.3%) | 7 (70%) | 0.556 | 0.842 |
| BA/PCA | 5 (10.4%) | 0 (0%) | 4 (14.3%) | 1 (10%) | 0.345 | 0.678 |
| *Collaterals* |  |  |  |  |  |  |
| Poor | 8 (18.6%) | 3 (33.3%) | 4 (16%) | 1 (11.1%) | 0.413 | 0.377 |
| Intermediate | 25 (58.1%) | 6 (66.7%) | 14 (56%) | 5 (55.6%) | 0.800 | 0.847 |
| Good | 10 (23.3%) | 0 (0%) | 7 (28%) | 3 (33.3%) | 0.158 | 0.167 |
| *EVT device* |  |  |  |  |  |  |
| Stent retriever alone | 7 (14.3%) | 3 (30%) | 4 (13.8%) | 0 (0%) | 0.333 | 0.102 |
| Aspiration alone | 27 (55.1%) | 2 (20%) | 18 (62.1%) | 7 (70%) | 0.037 | 0.042 |
| Stent retrieve and aspiration | 15 (30.6%) | 5 (50%) | 7 (24.1%) | 3 (30%) | 0.223 | 0.513 |
| *mTICI* |  |  |  |  |  |  |
| 2a | 4 (8%) | 2 (20%) | 2 (6.7%) | 0 (0%) | 0.297 | 0.177 |
| 2c | 1 (2%) | 0 (0%) | 1 (3.3%) | 0 (0%) | 0.734 | 1.000 |
| 3 | 45 (90%) | 8 (80%) | 27 (90%) | 10 (100%) | 0.551 | 0.238 |
| *Outcome measures* |  |  |  |  |  |  |
| mRS pre-stroke | 0 (0-2) | 0.5 (0-2.1) | 0 (0-1) | 0.5 (0-3) | 0.427 | 0.914 |
| Baseline NIHSS | 15.5 (8.9-22) | 16.5 (14.8-22.1) | 16 (8-22.1) | 12 (9.8-21) | 0.945 | 0.611 |
| 24-hour NIHSS | 8 (3-18) | 11.5 (3-17.1) | 7 (1-18.1) | 11 (4.3-21.3) | 1.000 | 0.985 |
| NIHSS at discharge | 4 (1-14.7) | 4 (1-15.3) | 3.5 (0-14.2) | 4 (2-16.4) | 0.997 | 0.969 |
| 3-month mRS | 3 (1-4.2) | 3 (1-6) | 2 (1-4) | 4 (2.7-4.7) | 0.265 | 1.000 |
| *Blood test parameters* |  |  |  |  |  |  |
| Delay onset to blood sample | 22.7 (12.4-28.8) | 22.5 (10.8-24) | 19.7 (12.5-29.3) | 22.8 (16.7-35.8) | 0.785 | 0.723 |
| Total WBCs (× 10^3/μL) | 9.2 (7.4-11.6) | 11.4 (8.9-15.1) | 8.3 (6.7-10.7) | 11.1 (8.3-13.2) | 0.010 | 0.630 |
| Neutrophils (× 10^3/μL) | 6.8 (5.5-9.1) | 7.4 (6.3-10.2) | 6.3 (4.3-8.2) | 8.7 (6.4-10.6) | 0.213 | 0.698 |
| Lymphocytes (× 10^3/μL) | 1.4 (1.1-1.8) | 1.2 (1-1.6) | 1.4 (1.1-1.8) | 1.4 (0.9-1.8) | 0.556 | 0.921 |
| Monocytes (× 10^3/μL) | 0.7 (0.6-0.9) | 0.7 (0.6-0.9) | 0.7 (0.6-0.8) | 0.7 (0.5-1.1) | 0.940 | 0.787 |
| Platelets (× 10^3/μL) | 187 (140-260) | 185 (114-272) | 183 (148-218) | 210 (125-377) | 0.875 | 0.395 |
| CRP (mg/L) | 18.1 (8.1-81.6) | 108.6 (70.7-171.9) | 9.8 (6.4-20.9) | 55.5 (15.3-75.8) | 0.000 | 0.012 |

Abbreviations – *ASPECTS, Alberta Stroke Program Early CT Score; BA, basilar artery; CRP, C reactive protein; EVT, endovascular thrombectomy, ICA, internal carotid artery; MCA, middle cerebral artery; mRS, modified Rankin Scale; NIHSS, National Institutes of Health Stroke Scale; PCA, posterior cerebral artery; TIA, transient ischemic attack; mTICI, modified treatment in cerebral ischemia; TOAST, Trial of ORG 10172 in Acute Stroke Treatment; VA, vertebral artery; WBCs, white blood cells. Numerical variables expressed as median (interquartile range), categorical variables expressed as numer (percentage).*

^+^ Indicates clinical features used for the matching procedure to select patients in the CE-I- group.

**Supplemental Table 2.** Details on the clinical presentation and the diagnosis of infective endocarditis.

|  | **Total (n=10)** |
| --- | --- |
| Delay from symptoms onset to diagnosis |  |
| < 1 month (acute) | 6 |
| 1-6 months (subacute) | 4 |
| *Valve type* |  |
| Native | 3 |
| Native with prosthetic ring annuloplasty | 2 |
| Bioprosthetic | 2 |
| Mechanical prosthetic | 3 |
| *Valve(s) involved* |  |
| Mitral | 4 |
| Aortic | 5 |
| Mitral and aortic | 1 |
| *Echocardiography findings** |  |
| Vegetations | 7 |
| Valve strands | 2 |
| Periprosthetic valve thickening | 1 |
| Perivalvular abscesses | 2 |
| *Timing of IE diagnostic relatively to stroke onset* |  |
| Before stroke onset | 4 |
| After stroke onset | 6 |
| *Timing of antibiotic therapy start relatively to stroke onset* |  |
| Before stroke onset | 6 |
| After stroke onset | 4 |
| *Clinical and radiological presentation* |  |
| Multifocal ischemic stroke lesions | 5 |
| Hemorrhagic transformation | 4 |
| Mycotic aneurysm | 0 |
| Systemic embolism | 6 |
| Other acute infectious complications^+^ | 2 |
| *Cardiac surgery* |  |
| Valve replacement by open surgery | 3 |
| None | 7 |

*Abbreviations – IE: infective endocarditis.*

**More than one finding could be present in a single patient. All patients had transthoracic echocardiograms (TTE) and/or transesophageal echocardiograms (TEE).*

*^+^Spondylodiscitis in one patient and septic shock in one.*

**Supplemental Figure 1.** Flowchart displaying the patients’ selection process applying inclusion and exclusion criteria of the study in the three participating centers.

**Supplemental Figure 2.** Flowchart for Managing Cerebral Thrombi in Suspected Infective Endocarditis undergoing Thrombectomy. Proposed flowchart for the handling and analysis of retrieved cerebral thrombi in cases of early suspected stroke due to endocarditis etiology, or when clinical doubts about endocarditis arise later during the diagnostic process. Abbreviations: Formalin-Fixed Paraffin-Embedded (FFPE), H&E, Hematoxylin and Eosin, PAS, Periodic Acid Schiff staining, GMS, Grocott's methenamine silver stain
