# Supplementary figures and images for: Cerebral thrombus analysis as a useful diagnostic tool for infective endocarditis in ischemic stroke patients
Source: Eur Stroke J. 2025 Feb 16;10(3):929–39. doi: 10.1177/23969873251320449 (PMC11831614; doi:10.1177/23969873251320449)

## Supplemental Figure 1

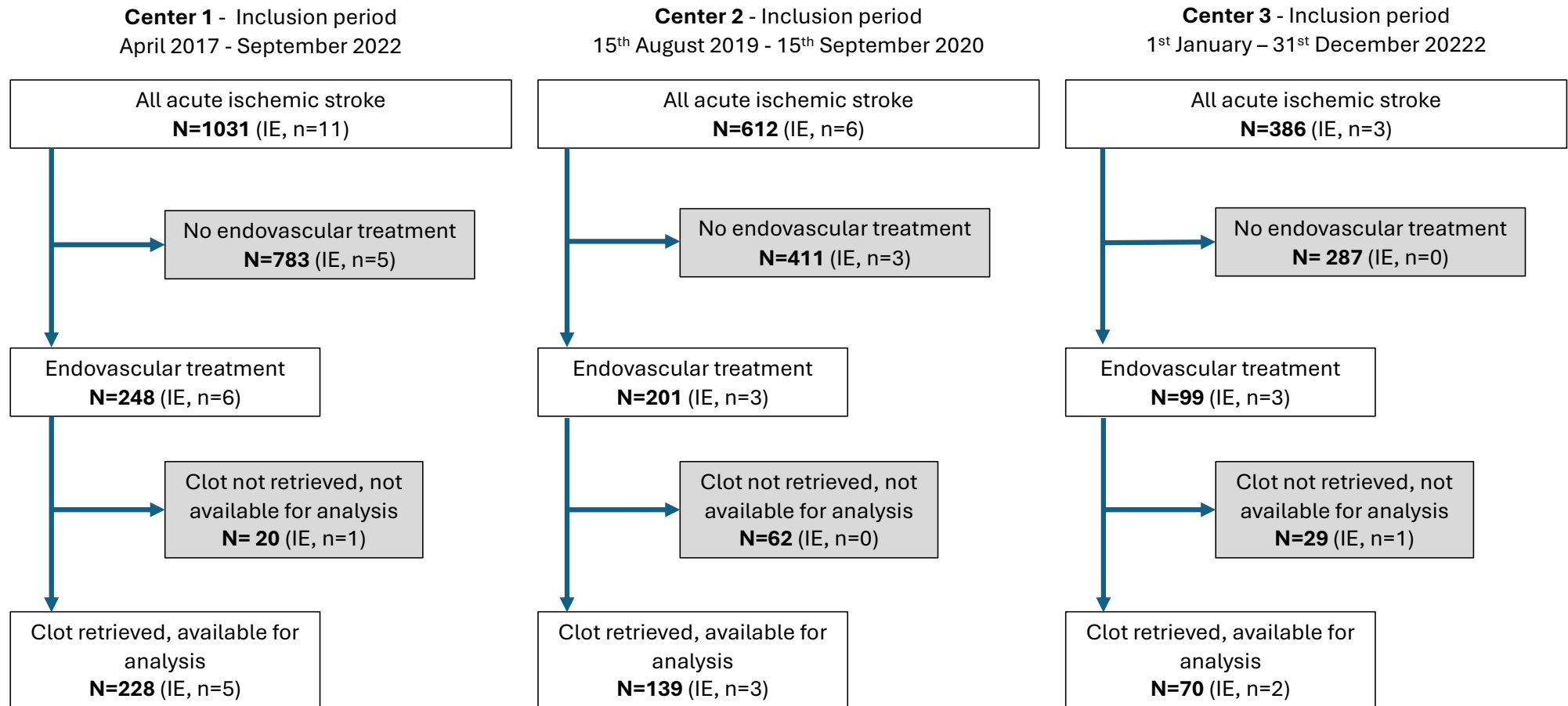

Supplement: sj-pdf-1-eso-10.1177_23969873251320449 – Supplemental material for Cerebral thrombus analysis as a useful diagnostic tool for infective endocarditis in ischemic stroke patients [file sj-pdf-1-eso-10.1177_23969873251320449.pdf]

**Supplemental Figure 2**

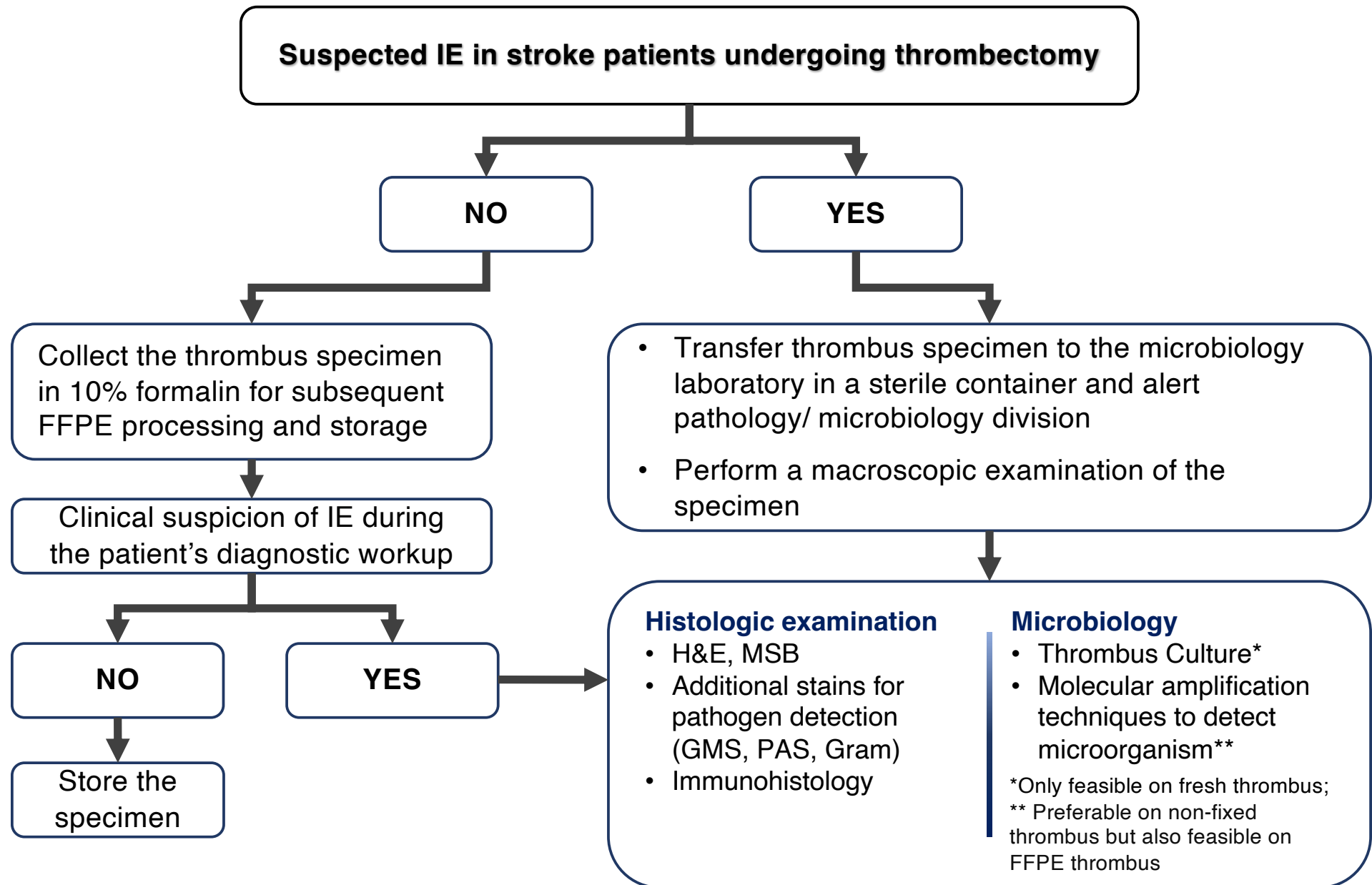

Supplement: sj-pdf-2-eso-10.1177_23969873251320449 – Supplemental material for Cerebral thrombus analysis as a useful diagnostic tool for infective endocarditis in ischemic stroke patients [file sj-pdf-2-eso-10.1177_23969873251320449.pdf]
